# Enhanced detection of gametocytes by magnetic deposition microscopy predicts higher potential for *Plasmodium falciparum *transmission

**DOI:** 10.1186/1475-2875-7-66

**Published:** 2008-04-25

**Authors:** Stephan Karl, Makindi David, Lee Moore, Brian T Grimberg, Pascal Michon, Ivo Mueller, Maciej Zborowski, Peter A Zimmerman

**Affiliations:** 1 Institute of Food Process and Bioprocess Engineering, University of Technology Dresden, 01062 Dresden, Germany; 2 Papua New Guinea Institute of Medical Research, P.O. Box 378, Madang, MP 511, Papua New Guinea; 3 Department of Biomedical Engineering/ND20, Lerner Research Institute, The Cleveland Clinic, 9500 Euclid Avenue, Cleveland, OH 44195, USA; 4 The Center for Global Health and Diseases, Case Western Reserve University, Wolstein Research Building, Room 4-125, 2103 Cornell Road, Cleveland, OH 44106-7286, USA

## Abstract

**Background:**

Aggregated haemozoin crystals within malaria-infected erythrocytes confer susceptibility of parasitized cells to a magnetic field. Here the utility of this method for diagnosis of human malaria is evaluated in a malaria-endemic region of Papua New Guinea (PNG).

**Methods and findings:**

Individuals with *Plasmodium falciparum *malaria symptoms (n = 55) provided samples for conventional blood smear (CBS) and magnetic deposition microscopy (MDM) diagnosis. Standard Giemsa staining and light microscopy was performed to evaluate all preparations. *Plasmodium falciparum *parasitaemia observed on MDM slides was consistently higher than parasitaemia observed by (CBS) for ring (CBS = 2.6 vs. MDM = 3.4%; t-test P-value = 0.13), trophozoite (CBS = 0.5 vs. MDM = 1.6%; t-test P-value = 0.01), schizont (CBS = 0.003 vs. MDM = 0.1%; t-test P-value = 0.08) and gametocyte (CBS = 0.001 vs. MDM = 0.4%; t-test P-value = 0.0002) parasitaemias. Gametocyte prevalence determined by CBS compared to MDM increased from 7.3% to 45%, respectively.

**Conclusion:**

MDM increased detection sensitivity of *P. falciparum*-infected, haemozoin-containing erythrocytes from infected humans while maintaining detection of ring-stage parasites. Gametocyte prevalence five-fold higher than observed by CBS suggests higher malaria transmission potential in PNG endemic sites compared to previous estimates.

## Background

Reliable malaria diagnosis including qualitative and quantitative detection of all malarial blood stages remains very important in the campaign against this disease, which is believed to kill one to two million children annually [1]. Comparing diagnostic approaches that have included conventional blood smear (CBS) light microscopy, enzyme-based rapid diagnostic tests (RDTs) and PCR-based strategies has revealed a greater complexity of malaria infection in individuals and population studies [2]. Furthermore, direct comparisons of these methods illustrate differences in sensitivity and specificity, cost and efficiency of diagnosis. These comparisons show that no single diagnostic approach can be used to satisfy all malaria diagnostic expectations. Whereas RDTs can be performed rapidly in the field or by highly sensitive and specific PCR-based processing in well-equipped laboratories, these methods lose the ability to evaluate parasite morphology that is exquisitely informative regarding parasite developmental stages of all the human malaria parasites. Quantitative evaluation of gametocytes in particular, could be useful in assessing potential for malaria transmission from humans to vector mosquitoes.

Recently, we developed a novel method, termed magnetic deposition microscopy (MDM) to enhance the detection of malarial blood stages exploiting their paramagnetic characteristics [3]. Malarial parasites convert erythrocyte haemoglobin to paramagnetic haemozoin [4]. The strong magnetic field of a permanent magnet is used to isolate infected red blood cells from a cell suspension and accumulate them on transparent polymer slides suitable for light microscopy. In preliminary studies on infected blood from splenectomized non-human primates we demonstrated that it was possible to increase the observable number of erythrocytic malarial blood stages by MDM at least 40-fold [3]. In this previous study, MDM yielded higher sensitivity than conventional thick blood smears with the clarity of a thin smear for diagnosis of all four human malaria parasite species. However, while trophozoites, schizonts and gametocytes were present in significantly higher numbers, we observed a deficiency of *Plasmodium falciparum *ring stages on the MDM slides.

Here we extend observations from our initial study to determine if it is possible to apply the MDM diagnosis of *P. falciparum *malaria in human infections through a field study in a malaria-endemic region of Papua New Guinea (PNG), conducted between March and June 2007. Moreover, as peripheral blood samples from acute symptomatic *P. falciparum *infections are characterized by a majority of ring-stage parasites that have yet to accumulate haemozoin, it was critical to evaluate the success in capturing these forms specifically.

## Methods

### Blood isolate collection and preparation

Blood samples were collected in 2007, over a period of two months during the wet season, at health centers in Madang Province in PNG. These samples were collected in partnership with in vivo drug trials where enrollment criteria excluded study participants with non-*P. falciparum *infections diagnosed by CBS. A total of 55 whole blood samples (1 ml) were collected from *Plasmodium*-infected individuals in heparin Vacutainer^® ^tubes (Greiner bio one GmbH, Kremsmuenster, Austria). Informed consent was obtained from all participants following protocols approved by the Medical Research Advisory Committee (MRAC) of PNG, the Institutional Review Board for Human Investigation at University Hospitals of Cleveland, and the International Centers for Tropical Disease Research Network/NIAID/NIH [5].

### Sample preparation and magnetic deposition microscopy

**Figure 1:**
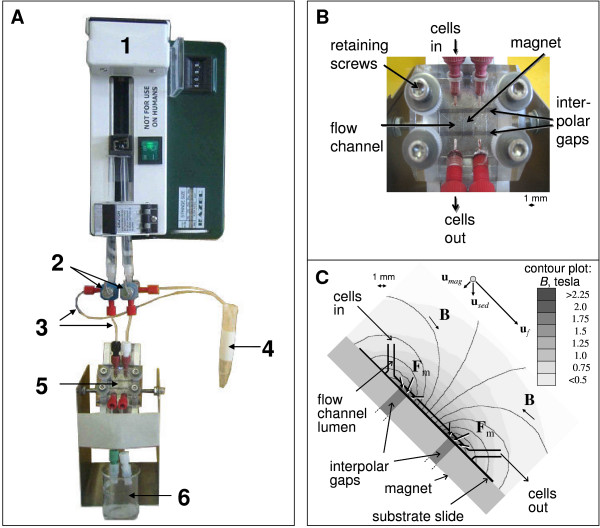
**Magnetic Deposition Microscopy (MDM) Assembly.** The MDM assembly (Panel A) is comprised of a syringe pump with adjustable flow rate (1), two valves (2) with outlets (3) to regulate flow and for air-free filling of the channels, the magnet with the flow chamber (5) and an outlet for the effluent cell suspension (6). PTFE tubing (4) is used to connect the different components. An enlarged view of the MDM device (Panel B) showing the flow channel formed between a top acrylic platen and bottom polymer slide, separated by a silicon rubber spacer. This assembly is fixed to a magnet by four retaining screws. The figure also shows portals through which cell suspensions enter and leave the flow chamber, and magnetic bars separated by two inter-polar gaps (dark gray). The schematic diagram of the MDM cell deposition section (Panel C) illustrates the force vectors acting on the paramagnetic cells (**F**_m_), and magnetic field lines (**B**). The field magnitude is indicated on the gray scale of the contour plot. Note paramagnetic cell concentration at the two interpolar gaps on the deposition substrate, as indicated by **F**_m _arrows. Also indicated are the cell velocity components: magnetic field-induced velocity, **u**_*mag*_, gravitational sedimentation, **u**_*sed*_, and the bulk carrier fluid flow velocity, **u**_*f *_(not to scale – see text for details).

Malaria magnetic deposition microscopy (MDM) has been described previously [3]. The system used in this study is depicted as a photograph in Figure 1A to 1C; key modifications included (a) addition of a second interpolar gap, and (b) positioning the MDM apparatus at a 45° angle. Empirical studies showed that positioning the apparatus at a 45° angle allowed blood samples to cover the entire width of the cell flow path (6.350 mm); blood flow covered a more limited surface area (approximately 1 mm) when the MDM device was perpendicular to the benchtop. The flow channel system was manufactured in the Rapid Prototyping Laboratory of the Cleveland Clinic. A medical grade rubber spacer (McMaster-Carr, Aurora, OH) with cutout defined the flow path of 6.350 × 0.254 mm (width × height). The permanent magnet was produced by Dexter Magnetics (Dexter Magnetic Technologies, Elk Grove Village, IL). The magnet assembly contained two 1.27 mm wide interpolar gaps separated by a center pole piece of 7.0 mm width, where the cell flow path and the high magnetic flux density deposition zones were located at the edges of each interpolar gap (predicted, maximum, magnetic energy density gradient was 4.41 T^2^/mm and the maximum flux density was 2.27 T). Figure 1C illustrates the flow chamber, areas of magnetic deposition, and magnetic, gravitational and drag forces.

The magnet system was assembled in a laminar flow hood. The apparatus flow channel was flushed (10 ml/hr for 10 min) and slides were treated with 96% ethanol before every experiment to avoid contamination. The system was treated by UV light between experiments to maintain sterility. Ethanol was then replaced by 1× phosphate buffered saline, pH 7.4 (PBS) at a flow rate 10 ml/hr for 10 min. Blood samples were washed in culture medium (RPMI from Invitrogen/Gibco, Carlsbad, CA, supplemented with 25 mg/ml HEPES, 2 mg/ml sodium bicarbonate, 5% albumax II) three times by centrifugation (BD Allegra centrifuge, 500 × g, 8 min) and resuspension. The majority of the white blood cells were taken out by removing the buffy coat above the red cell pellet each time prior to resuspension. Packed red blood cells (rbc) (20 μl) were suspended in 1 ml of the same pre-warmed culture medium; final cell concentration approximately 10^8 ^rbc/ml. The 1 ml cell suspension was injected into the magnet assembly using a single syringe pump (model R99-EJM, Razel Scientific Instruments, St Albans, VT) at 8.3 μl/min via PTFE tubing with an ID of 0.254 mm (Upchurch Scientific, Oak Harbor, WA), as illustrated in Figure 1. As infected rbcs were captured by the magnetic field, they deposited on the 127 μm thick polymer slide. After the cell suspension had passed across the two deposition zones, fluid was removed from the flow chamber with air using the syringe pump at 4.13 μl/min. The polymer slides were removed from the magnet assembly and mounted onto glass microscope slides. For comparison, a 2 μl blood aliquot of each patient blood sample was used for preparing thick and thin CBS according to standard protocols [6]. Both MDM and CBS slide preparations were stained with a 4% Giemsa solution for 9 min and examined by light microscopy under a 100× oil-immersion objective.

The cell deposition on the substrate slide was the result of these distinct phenomena: sedimentation due to gravity, cell entrainment in the carrier fluid flow, and the magnetic field-induced retention. Based on the physical characteristics of the cell, those of the carrier fluid, the geometry and the dimensions of the flow channel, the volumetric flow rate of the carrier fluid and the properties of the magnet assembly, the cell velocity components due to those different phenomena were calculated and presented as a vector diagram in Figure 1. The magnitudes of the cell velocity components were as follows: the magnetic field-induced velocity, *v*_*mag*_, varied strongly with the cell position, with the average value of 2.5 μm/s (peak value of 27.4 μm/s); the cell sedimentation velocity, *v*_*sed *_= 3.3 μm/s; and the cell entrainment velocity in the bulk flow, *v*_*f *_= 133 μm/s. Thus the results show that the dominant velocity component is due to cell the entrainment in the bulk fluid flow, and that the average magnetic field-induced velocity is comparable to the cell sedimentation velocity. Since the sedimentation is a significant component of the cell velocity, and the entrainment velocity decreases rapidly in the proximity of the substrate slide due to a parabolic velocity profile of the laminar flow, the results suggest that the erythrocytes travel close to the substrate slide surface. The model predicts that the majority of the erythrocytes roll over an inclined surface of the slide (Figure 1) as they approach the magnetic capture region at the interpolar gaps, and that they may be in contact with the slide surface for the entire length of the flow channel. This suggests increased capture of weekly magnetic erythrocytes and an occasional erythrocyte capture unrelated to the action of the magnetic field.

### Slide evaluation

Prior to analysis of MDM preparations, the CBS slides were evaluated for parasite species, developmental stage distribution and parasitaemia (by two independent microscopists) by counting 200 microscope fields, or approximately 40,000 rbc at 1,000× magnification (Olympus BX 241 microscope, 100× oil immersion objective) or 100 malaria parasites, whichever came first. In cases of low parasitaemia of any blood-stage form, infected rbc were qualitatively assessed by scanning the entire thick and thin films, or the equivalent of four μl of infected blood. For MDM slides, since the magnetic field was inhomogeneous, the parasitaemia was evaluated in the zones where the highest field gradient was present, and therefore, the highest deposition rate would be expected (compare Figure 1C). Random MDM fields were scanned in the deposition zone until at least 1,000 rbc had been counted. The entire MDM slides were also scanned for the existence of all developmental stage forms. Developmental stage deposition was documented on photomicrographs captured with a Nikon Eclipse 50i microscope and camera (Nikon Corporation, Japan).

### DNA template preparation

DNA was extracted from whole blood (200 μL) using protocols recommended for the QIAamp 96 DNA Blood Kit (QIAGEN, Valencia, CA).

### PCR amplification and *Plasmodium *species-specific LDR-FMA

All methods for the PCR amplification of small sub-unit rRNA target sequences and *Plasmodium *species-specific detection by ligase detection reaction/fluorescence microsphere assay (LDR-FMA) have been described in detail [7,8] As *these studies *were conducted in PNG in regions where all four species of human malaria parasites (*P. falciparum, P. vivax, Plasmodium malariae and Plasmodium ovale*) are endemic, molecular diagnostic experiments were used to validate the findings of microscopy performed by CBS and MDM light microscopy. The presence or absence of a certain species by LDR-FMA was compared to CBS and MDM.

### Statistical analysis of CBS vs MDM comparisons

The resulting pairs of CBS and MDM slides were analysed for each isolate. From the parasitaemias (*p*) observed with the two different methods the fold-increase in sensitivity with MDM (*I*_*S*, *MDM*_) relative to CBS was calculated with the following equation.

$$I_{S,MDM}=\frac{p_{MDM}}{p_{CBS}}$$

The increase in sensitivity was calculated separately for each of the asexual blood stages (ring, trophozoite, schizont) and for gametocytes. The mean increase in sensitivity for each developmental blood stage was calculated from data for all 55 single experiment enrichments. The overall *I*_*S*, *MDM *_was the sum of mean developmental stage increases in sensitivity for 55 experiments. Similarly, mean stage parasitaemias and overall parasitaemia were calculated based on CBS and MDM counts. The difference in mean parasitaemias between the two methods was tested (Student t-test) at a significance level of 0.05, *N *= 55. The fold increase in sensitivity was considered significant if the difference in mean parasitaemias, *p*_*MDM *_and *p*_*CBS*_, entered in the expression for *I*_*S*.*MDM*_, above, was significant. The prevalence of gametocytes and schizonts in the studied population was also analysed by comparing the frequency of their occurrence as detected by CBS and MDM.

## Results

### Assessing MDM field diagnosis of *P. falciparum *infection

**Figure 2:**
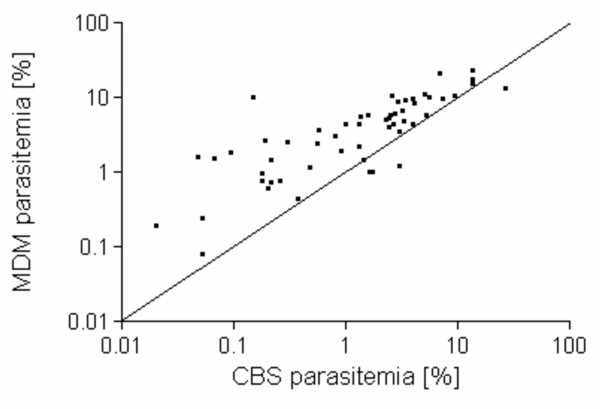
**Comparison of paired CBS and MDM results from *P. falciparum*-infected study participants.** MDM parasitaemia (Y-axis) is compared to CBS parasitaemia (X-axis) for paired of analysis of **55 ***P. falciparum*-infected blood samples. The line bisecting the graph identifies where points would fall if identical results were observed between the two diagnostic methods (*I*_*S*, *MDM *_> 1).

**Figure 3:**
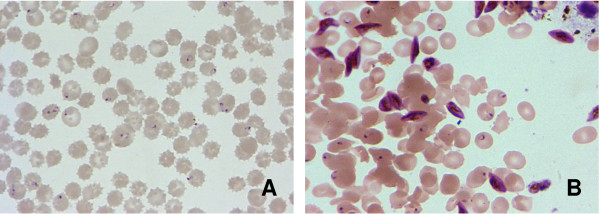
**Paired CBS and MDM preparations from one study participant.** Panel A shows the CBS preparation, Panel B shows the MDM preparation; both panels show the same surface area of an individual microscope field (1000×). The CBS preparation shows 19 ring-stage parasites in the thin smear. The total number of cells in the field is 125; overall percentage of *P. falciparum*-infected cells in Panel A is 15.2% (19/125). The MDM preparation shows 16 ring-, and 16 gametocyte-stage parasites in the thin smear. The total number of cells in the field is 131; overall percentage of *P. falciparum*-infected cells in Panel B is 24.4% (32/131).

**Figure 4:**
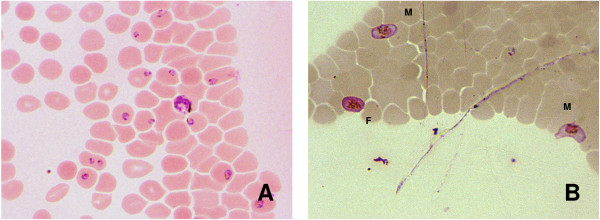
**MDM enrichment of *P. falciparum *developmental stages from peripheral blood.** Examples of MDM microscopy images (Panel A) show mixed stages including rings, trophozoites and schizonts. Panel B illustrates capture of male (M) microgametocytes and a female (F) macrogametocyte.

Previous demonstration of MDM enriched capture of infected rbcs from *Plasmodium *species-infected non-human primates [3] suggested that this same approach might be useful in diagnosis of malaria parasite infection in human blood samples. The summary of paired CBS and MDM results from 55 *P. falciparum*-infected study participants (Figure 2) showed that this strategy enriched capture of parasitized cells from infected individuals in field-based studies, as indicated by the majority of data points rising above the line representing identical performance of methods (*I*_*S*, *MDM *_> 1) in more than 90% of cases. Overall, MDM resulted in a significantly enriched capture of infected rbcs compared to the representation of uninfected and infected rbcs observed on CBS slides (infected rbcs by CBS = 3.19%, MDM = 5.46%; Student t-test P-value = 0.016). Figure 3A and 3B illustrate differences between paired CBS and MDM preparations from one study participant as a significant increase in the number of parasitized erythrocytes observed in individual microscopy fields. Of particular interest was the notable increase in detection of gametocytes. Additional examples of MDM microscopy images (Figure 4A) show mixed stages including rings, trophozoites, and schizonts; Panel 4B illustrates capture of male (M) microgametocytes and a female (F) macrogametocyte.

**Figure 5:**
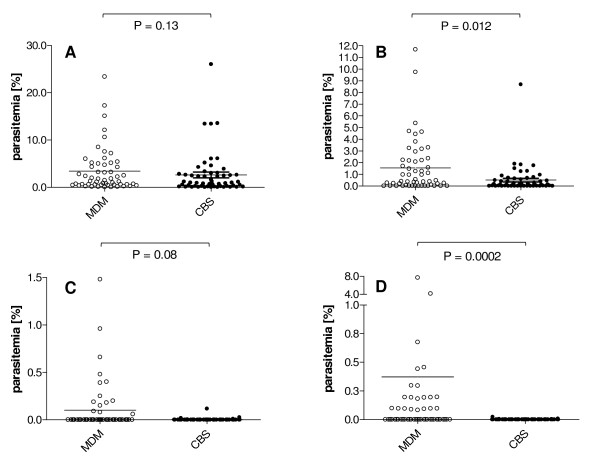
***P. falciparum* developmental stage parasitaemias observed by CBS and MDM.** CBS (gray) and MDM (blue) parasitaemias for ring (Panel A), trophozoite (Panel B), schizont (Panel C) and gametocyte (Panel D) stages for 55 *P. falciparum*-infected individuals.

We further evaluated the CBS and MDM slides to determine whether the observed enrichment by MDM applied to all *P. falciparum *developmental blood stages (Figure 5). Interestingly, we observed no significant difference in capture of ring or trophozoite stage parasites for CBS vs. MDM preparations (mean ring stage parasites – CBS = 2.61% [st dev ± 4.41%], MDM = 3.42% [st dev ± 4.29%], Mann-Whitney t-test P-value = 0.13; mean trophozoite stage parasites – CBS = 0.51% [st dev ± 1.23], MDM = 1.55% [st. dev. ± 2.67], Mann-Whitney t-test P-value = 0.012). In contrast to these observations for early parasite developmental stages, we observed significant increases in the capture of *P. falciparum *schizont and gametocyte stages by MDM (mean schizont stage parasites – CBS = 0.003% [st. dev. ± 0.08], MDM = 0.1% [st. dev. ± 0.3], Mann-Whitney t-test P-value = 0.08; mean gametocyte stage parasites – CBS = 0.001% [st. dev. ± 0.003], MDM = 0.37% [st. dev. ± 1.4], Mann-Whitney t-test P-value = 0.0002).

**Figure 6:**
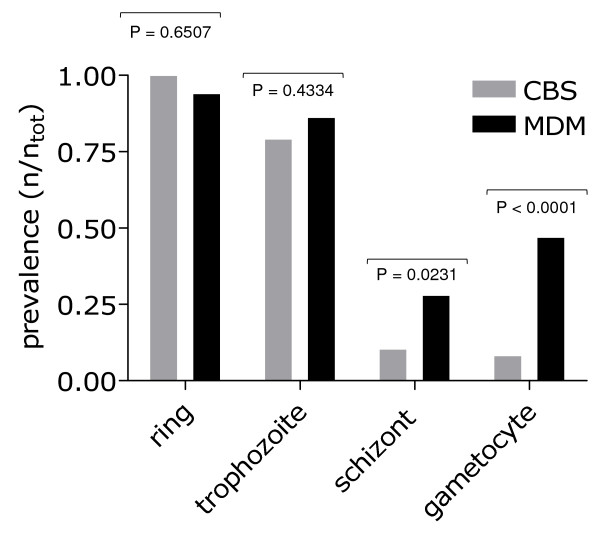
Prevalence of *P. falciparum *developmental stages observed by CBS and MDM. CBS (gray) and MDM (blue) prevalence for ring, trophozoite, schizont and gametocyte stages for 55 *P. falciparum*-infected individuals.

In light of these observations focused on the enriched capture of parasitized rbc by MDM vs. CBS, we evaluated the prevalence at which each of the *P. falciparum *developmental blood stages were observed by these two preparations within the 55 study participants (Figure 6). Consistent with the results reported above, we found that the prevalence of ring and trophozoite stage parasites was similar in CBS and MDM preparations (prevalence of ring stage parasites – CBS = 96% [53/55], MDM = 95% [52/55]; trophozoite stage parasites – CBS = 82% [45/55], MDM = 87% [48/55]). The prevalence of schizont developmental stages was 2.7-fold higher by MDM vs. CBS, and the prevalence of gametocytes was 6.2-fold higher by MDM vs. CBS (45% [25/55] vs 7.3% [4/55]).

### Correlating MDM and LDR-FMA species diagnosis

Validation of the MDM results was performed by comparison with DNA-based diagnosis using a *Plasmodium *species-specific LDR-FMA strategy [8] Our comparison of LDR-FMA and MDM diagnoses showed that there was no discordance suggesting that any species other than *P. falciparum *was observed by the molecular diagnostic technique.

## Discussion

Our study here demonstrates that MDM [3] successfully detects *P. falciparum *in individuals experiencing symptoms of malarial illness in Papua New Guinea. Although it was surprising that we did not detect non-falciparum species by MDM in this region where we have consistently observed all four human *Plasmodium *parasite species in past studies [7,9,10], patient samples were obtained from studies evaluating antimalarial drugs where enrollment criteria avoided complex infections. Moreover, the species-specific detection of *P. falciparum *only, by our recently developed post-PCR LDR-FMA assay [8] for all four human *Plasmodium *species parasites, lends strong evidence that study participants were not infected with non-*falciparum *species.

Modifications to the MDM device used in this study offer conveniences that facilitate transportation, set up and operation for day-to-day use. More importantly, the introduction of a second interpolar gap increased the overall surface area of the magnetic gradient in the current MDM device. Of additional interest, we also observed that positioning the device at a 45° angle to the bench-top surface led to spreading of the blood volume across the entire surface of the sample flow path to increase the number of cells exposed to the magnetic field per blood volume assayed. Future experiments will be needed to determine how combined interactions between magnetic field, gravity and surface tension influence the flow of *Plasmodium *infected and uninfected cells through the MDM device. Of practical importance here, these modifications have contributed to our observations reporting routine capture of *P. falciparum *ring-stage parasites on MDM microscope slides at levels consistent with CBS.

Although our earlier work demonstrated that gametocytes were susceptible to MDM capture [3], our study here confirms these results in a malaria-endemic field setting using infected human blood samples, and presents observations that are integral to gametocyte biology and transmission of *P. falciparum*. This includes evidence that MDM captures both male and female gametocytes, and that many individuals carry numbers of mature gametocytes in blood volumes within the range ingested during female mosquito blood meals. Numerous recent studies have reported on gametocyte prevalence that occurs at, or below, the limits of CBS detection [11-18]. The majority of these studies have relied on the increased sensitivity of reverse transcriptase-PCR methods to detect mRNA expressed specifically during gametocyte stages of development [11-13,15-18]. While these approaches have provided important insight regarding a critical component of *P. falciparum *diagnosis, they require laboratory-based procedures that are difficult to perform in a timely fashion in close proximity to the field settings where human to mosquito transmission occurs. Furthermore, quantitative assessments of gametocyte levels by nucleic acid based methods will only provide a rough approximation of mature gametocyte numbers [19,20]. Interestingly, the increased prevalence at which gametocytes were observed in patient samples by MDM vs. CBS in this study (6.2-fold increase; CBS gametocyte prevalence similar to previous PNG-based studies [3.3–13%] [21,22]) is similar to findings comparing nucleic acid-based diagnostic methods vs. CBS [11,13,16,18].

As the level at which gametocytes become optimally infectious to mosquitoes is not clear, or may vary based on endemic seasonality, or local vector species preferences [23-25], it is important to improve the capacity for identifying and enumerating gametocytes in human blood samples. A number of field observations now suggest that humans are infectious to mosquitoes even at gametocyte levels below detection limits of CBS light microscopy [14,16,17,26]. In order to evaluate the impact of current insecticide-treated bed net, antimalarial drug or vaccine control programs, it will be necessary to enumerate accurately mature gametocyte densities circulating in the blood of infected people. As MDM diagnosis provides counts of mature male and female gametocytes within in given blood volume, this diagnostic strategy may help to clarify the relationship between gametocyte density and productive infection of mosquitoes.

In future studies it will be important to expand direct comparisons of malaria diagnosis performed by MDM, CBS (against, 200, 500 and 1000 leukocytes), rapid diagnostic tests (RDTs), and PCR-based techniques targeting *Plasmodium *species sequences. An overall comparison of diagnostic strategies will provide opportunity to determine if this new microscopy-based method is capable of delivering sensitivity rivaling PCR, along with the morphological resolution of a thin smear blood film. Also, as PCR-based diagnostic approaches are far superior to other diagnostic strategies for *Plasmodium *species identification, it will be important to evaluate the concordance of species diagnosis between MDM and PCR. Direct comparison with the antigen capture-based RDTs would also apply continuing pressure to improve performance of products that are currently expensive, frequently deliver lower sensitivity than CBS [27], and are susceptible to reduced sensitivity by amino acid sequence polymorphism. Moreover, it is important that all of the diagnostic strategies are forced to compete so that durable, rapid, inexpensive and accurate malaria diagnostic tools are available in settings where the disease is most difficult to control.

Finally, whereas the study performed here compared CBS and MDM diagnosis on people reporting to a health center with malaria symptoms, it will be important to perform a similar comparison study among a series of asymptomatic individuals. A study of this nature will enable evaluation of MDM on a large number of people who are likely to carry very low levels of parasitaemia. As our past studies have identified frequent mixed *Plasmodium *species infections in cross-sectional surveys, we would expect MDM to detect mixed species infections in asymptomatic individuals more often than CBS. Further, gametocytes are thought to be most prevalent in individuals who are experiencing clinical symptoms, and less common in individuals who have developed acquired immunity against *Plasmodium *species parasites. A survey of asymptomatic individuals, including MDM diagnosis, will provide new insight regarding the overall prevalence of gametocytes in malaria-endemic populations. This may reveal important new reservoirs contributing to malaria transmission and further refine malaria control strategies.

## Abbreviations

PNG: Papua New Guinea; CBS: conventional blood smear; MDM: magnetic deposition microscopy; LDR-FMA: ligase detection reaction fluorescent microsphere assay.

## Authors' contributions

SK contributed to the experimental and epidemiologic designs, performed all sample preparation, processing and evaluation of CBS and MDM slides. BTG contributed to the experimental design. MD, PM and IM contributed to coordination of the field studies in PNG. SK, LM and MZ designed the MDM device. PAZ developed the Plasmodium species PCR-based diagnostic assay. MZ and PAZ supervised the overall project. SK, MZ and PAZ wrote the manuscript, with assistance from their collaborating authors. All authors read and approved the final manuscript.
